# Kinetics and durability of transgene expression after intrastriatal injection of AAV9 vectors

**DOI:** 10.3389/fneur.2022.1051559

**Published:** 2022-11-14

**Authors:** Bradley S. Hollidge, Hayley B. Carroll, Randolph Qian, Madison L. Fuller, April R. Giles, Andrew C. Mercer, Olivier Danos, Ye Liu, Joseph T. Bruder, Jared B. Smith

**Affiliations:** Research and Early Development, REGENXBIO Inc., Rockville, MD, United States

**Keywords:** AAV, promoter, durability, striatum, intraparenchymal, episome

## Abstract

Understanding the kinetics and durability of AAV-mediated transgene expression in the brain is essential for conducting basic neuroscience studies as well as for developing gene therapy approaches for CNS diseases. Here, we characterize and compare the temporal profile of transgene expression after bilateral injections into the mouse striatum of rAAV9 encoding GFP under the control of either a ubiquitous promoter (CAG), or the neuron-specific human synapsin (hSyn) and CamKII promoters. GFP protein expression with the CAG promoter was highest at 3 weeks, and then decreased to stable levels at 3 and 6 months. Surprisingly, GFP mRNA levels continued to increase from 3 weeks to 3 months, despite GFP protein expression decreasing during this time. GFP protein expression with hSyn increased more slowly, reaching a maximum at 3 months, which was equivalent to protein expression levels from CAG at that time point. Importantly, transgene expression driven by the hSyn promoter at 6 months was not silenced as previously reported, and GFP mRNA was continuing to rise even at the final 6-month time point. Thus, hSyn as a promoter for transgene expression demonstrates long-term durability but may require more time after vector administration to achieve steady-state levels. Because CAG had the highest GFP protein expression in our comparison, which was at 3 weeks post administration, the early kinetics of transgene expression from CAG was examined (1, 2, 5, and 10 days after injection). This analysis showed that GFP protein expression and GFP mRNA increased during the first 3 weeks after administration. Interestingly, vector DNA rapidly decreased 10-fold over the first 3 weeks following injection as it assembled into stable circular episomes and concatemers. Surprisingly, the processing of vector genomes into circular episomes and concatemers was continually dynamic up to 3 months after injection. These results provide novel insight into the dynamic processing of vector genomes and promoter-specific temporal patterns of transgene expression in the brain.

## Introduction

Adeno-associated virus (AAV), a single-stranded DNA virus of the *Parvoviridae* family, has been harnessed as a viral vector to deliver transgenes to the brain for therapeutic use as well as for neuroscience research. With the maturation of the gene therapy field, practically every aspect of recombinant AAV (rAAV) vectors are being engineered, giving rise to a plethora of capsid variants (in addition to the naturally occurring serotypes) as well as promoters, enhancers and mRNA modulatory motifs to control transgene expression (1). One primary objective of capsid engineering is improving the efficiency of rAAVs to cross the blood-brain barrier after intravenous administration (2–4). However, systemic rAAV delivery to target the brain requires high doses because only a fraction of the vectors penetrates the blood-brain barrier to access the central nervous system (CNS) (2, 5, 6). Such large doses increase immune responses to the vector and can lead to hepatotoxicity as well as complement activation (7, 8). Furthermore, global biodistribution requires mitigating transgene expression in non-target tissues *via* cell-type specific promoters. Additionally, about 30–60% percent of the population has pre-existing neutralizing antibodies to specific AAV serotypes (9), which currently precludes the utility of these serotypes for systemic rAAV gene transfer in this subset of patients.

An alternative approach to systemic delivery is direct injection of rAAV into the brain parenchyma. This method requires less vector to achieve therapeutic transgene levels, reduces peripheral tissue distribution, allows for more precise targeting of specific brain regions, lowers the immune response to the vector and still provides efficient transduction in the presence of clinically-relevant levels of neutralizing antibodies (10–13). However, intraparenchymal delivery in the brain is a highly invasive procedure requiring neurosurgery and likely limiting treatment to a one-time administration. Therefore, understanding the duration of transgene expression is vital. Apropos, a previous study suggested that transgene (GFP) expression in the cerebellum driven by the human synapsin (hSyn) promoter is repressed or inactivated by 22 weeks following intravenous administration to neonatal rats (14). Therefore, determining if the hSyn promoter behaves similarly in the adult brain *via* intraparenchymal delivery is important to the development of gene therapies for CNS diseases.

Understanding transgene expression in the CNS is also pertinent to systems neuroscience research, which frequently employs AAV as an experimental tool. For example, utilizing rAAVs encoding fluorescent proteins allows for anatomical mapping of neural circuitry to trace axonal connections between brain regions (15). In addition, optogenetics allows for the manipulation of neuronal activity through light-activated ion channels, i.e., channelrhodopsins, that are expressed in a cell-type specific manner *via* Cre driver lines by rAAV driven Cre-dependent expression, which allows functional probing of neural circuits in awake, behaving animals (16). Furthermore, designer receptors exclusively activated by designer drugs (DREADDs), which are genetically modified G-protein-coupled receptors that can be specifically activated by a synthetic ligand, can be delivered by rAAVs in cell-type specific manners *via* cell-type specific promoters (17). In the CNS, the neuron-specific promoters, hSyn and CamKII, have been shown to drive reliable and robust expression that restricts primarily to neurons (18–20). In addition, the CAG promoter has been used to drive transgene expression within the CNS.

Understanding the expression kinetics of commonly used promoters like CAG, hSyn, and CamKII are important for experimental design and interpretation. To achieve successful and durable transgene expression from rAAVs require multiple steps. After entering the cell, the single-stranded, linear vector genome is released in the nucleus where genome processing occurs. Here, the single-stranded AAV DNA is transformed into double-stranded, linear genomes. These double-stranded, linear genomes can then become stable monomeric and/or concatemeric circular episomes through homologous recombination or non-homologous end-joining (21). These circular episomes are the predominant DNA species that is associated with stable, long-term expression of transgenes as has been demonstrated in the lung, liver and muscle (22–25). However, the formation of stable, circular episomes has not been examined in the brain after rAAV administration. Here, intraparenchymal delivery to the adult mouse striatum of rAAV9 encoding GFP driven by the CAG, hSyn, or CamKII promoters was examined to understand the kinetics and durability of transgene expression as well as circular episome formation.

## Materials and methods

### Adeno-associated viral vectors

rAAV9-CAG-GFP, rAAV9-hSyn-GFP and rAAV9-CamKII-GFP were produced at Vigene (Rockville, MD, USA). The promoter sequences used are listed in Supplementary Table S1. All vectors have the same GFP sequences, 3′ UTR and poly (A) tail.

### Mice

All animal experiments were conducted under an Institutional Animal Care and Use Committee (IACUC)-approved protocol in compliance with the Animal Welfare Act, PHS Policy, and other Federal statutes and regulations relating to animals and experiments involving animals. The facility where this research was conducted (CL Laboratory LLC) is accredited by the AAALAC International and the experiments were conducted in accordance with the National Research Council “Guide for the Care and Use of Laboratory Animals.”

**Figure 1:**
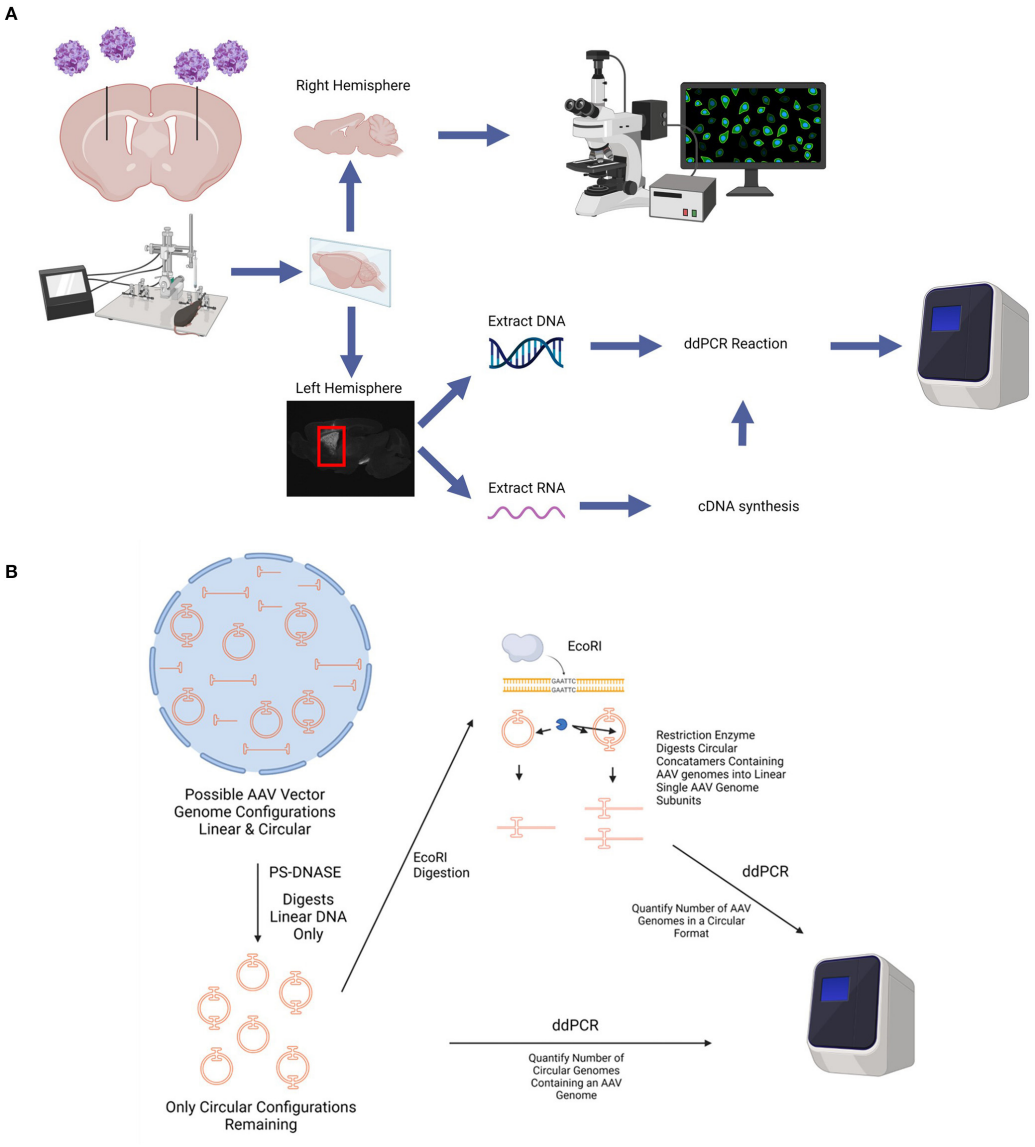
Diagram of *in vivo* experimental paradigm. **(A)** rAAV9 encoding GFP under the promoters CAG, hSyn, or CamKII were stereotaxically injected into the striatum of adult (at least 6 weeks of age), female C57BL/6 mice bilaterally. At various time points after AAV administration, mice were euthanized, perfused with saline, and the brains were collected. The right hemisphere of the brain was fixed in 4% PFA, cryoprotected in a 30% sucrose solution, and sectioned using a microtome for histological analyses. The striatum from the left hemisphere was micro-dissected and frozen. DNA and RNA was extracted from each striatum. cDNA was synthesized from the RNA. The number of AAV genome copies per cell and GFP transcripts were determined using ddPCR. **(B)** Diagram depicting circular AAV genome quantification assay. Total DNA is extracted from tissues and treated with PS-DNASE which digests all linear AAV genomes. Only circular AAV genomes are left behind. Quantification of this population of AAV genomes on ddPCR gives number of circular AAV genomes containing an AAV genome. This does not account for different sized AAV concatemers due to an end point measurement nature of ddPCR and thus measures total number of circular DNA with an AAV genome. To quantify number of AAV genomes in a circular format, the PS-DNASE digested samples are further digested with ECORI. ECORI cuts a single time between the promoter and EGFP sequences. Concatemers with multiple AAV genomes will be digested into multiple AAV linear genome subunits. Quantification of this population of AAV genomes with ddPCR will accurately give number of AAV genomes in a circular format. Created using images from BioRender.com.

Adult (>6 weeks of age) female C57BL/6J mice (*n* = 4 per group) were maintained on a 12 h light/dark cycle. The mice were provided food and water *ad libitum*, and group housed. A schematic of the experimental workflow for *in vivo* studies is shown in Figure 1.

### Stereotaxic injections into the striatum

Mice were anesthetized with ketamine (100 mg/kg)-xylazine (10 mg/kg) and then secured in a stereotaxic frame (Stoelting Co., Wood Dale, IL, USA). Holes were drilled into the skull using a dental drill. A 32G needle affixed to a 10 uL Hamilton syringe (Hamilton Company, Reno, NV, USA) was inserted into the brain through the craniotomies at coordinates corresponding to the striatum (in millimeters; anteroposterior (AP) +0.25, mediolateral (ML) ±2.00, dorsoventral (DV) −2.50 relative to bregma). In each striatum, 2 μL of vector (1.2 × 10^10^ vg per hemisphere) was injected at a constant rate of 0.2 μL per min using a syringe pump (Stoelting Co., Wood Dale, IL, USA). The needle was left in place for 2 min to minimize upward flow of solution. The scalps were then sutured, and the mice were allowed to recover. Buprenorphine SR was administered for post-operative analgesia.

### Tissue collection

Mice were heavily anesthetized with ketamine/xylazine and were then transcardially perfused with normal saline (0.9% NaCl) until the liver was clear. The whole brain was removed, and the right hemisphere was immersed in 4% paraformaldehyde overnight followed by cryoprotection in a 30% sucrose solution. The striatum from the left hemisphere was micro-dissected and frozen at −80°C for subsequent nucleic acid extraction.

### Immunohistochemistry

Brain hemispheres were sectioned at 50 μm on OCT compound (Tissue Tek, Torrance, CA, USA) using a Leica freezing microtome and slices were stored in tissue collection solution (0.045 M phosphate buffer, 30% ethylene glycol, and 25% glycerol). Sections were washed for 15 min in TBS thrice and then blocked for 1 h in TBS with 3% normal horse serum (Vector Laboratories, Newark, CA, USA) and 0.25% Triton-X (TBS+). The sections were then incubated with primary antibodies in TBS+ for 48 h at 4°C. After a washing in TBS twice for 15 min and then TBS+ for 30 min, sections were incubated with secondary antibodies at room temperature for 2 h. Sections were incubated with DAPI (Invitrogen, Waltham, MA, USA) at 5 μg/mL for 5 min, washed in TBS thrice for 15 min and then mounted on slides and coverslipped with mounting media (ProLong Gold anti-fade). The primary antibodies used were chicken anti-GFP (#NB100-1614, Novus Biologicals, Centennial, CO, USA) at 1:1,000; rabbit anti-Iba1 (#E404W, Cell Signaling Technologies, Danvers, MA, USA) at 1:500; mouse anti-NeuN (#ab104224, Abcam, Cambridge, MA, USA) at 1:1,000; rabbit anti-Olig2 (#ab109186, Abcam, Cambridge, MA, USA) at 1:250, and rabbit anti-S100β (#ab52642, Abcam, Cambridge, MA, USA) at 1:1,000. The secondary antibodies were all used at 1:250 and were as follows: donkey anti-chicken IgY Alexa Fluor 488 (Invitrogen, Waltham, MA, USA), donkey anti-mouse IgG Alexa Fluor 555 (Invitrogen, Waltham, MA, USA), and donkey anti-rabbit IgG Alexa Fluor 647 (Invitrogen, Waltham, MA, USA).

### Microscopy

Images for luminance quantification were taken using a LEICA M205 FCA at 1X. A 500 ms exposure was used to measure the native fluorescent luminance from GFP based on the versatile dynamic range revealed in an exposure series analysis of 100, 500, and 1,000 ms (Supplementary Figure S1). GFP luminance analysis of striatum was performed on ImageJ with an ROI restricted to the expression in striatum. A Zeiss LSM 900 Airyscan 2 confocal microscope was used to collect images for colocalization of GFP with cell markers labeled with immunohistochemistry. For quantification of colocalization, cell counts of images taken with 20X and 63X objectives were quantified using ImageJ.

### ddPCR

The striatums from the left hemispheres were homogenized in lysis buffer RA1 (Macherey-Nagel, Allentown, PA, USA) with 1% β-mercaptoethanol using a Precellys Evolution tissue homogenizer (Bertin Technologies, Rockville, MD, USA). DNA was extracted from an aliquot of the tissue homogenate using the DNeasy Blood & Tissue Kit (Qiagen, Germantown, MD, USA) following manufacturer's instructions. RNA was extracted from a different aliquot of tissue homogenate using the NucleoSpin RNA Kit (Macherey-Nagel, Allentown, PA, USA). RNA samples had an additional DNase treatment (DNA-free DNA Removal Kit [Invitrogen, Waltham, MA, USA]) to remove all DNA. cDNA was synthesized from the extracted RNA using the High Capacity cDNA Reverse Transcription Kit (Applied Biosystems, Waltham, MA, USA) following manufacturer's instructions. DNA or cDNA was combined with Naica multiplex PCR mix, as well as probes and primers for each target (GFP, mouse glucagon, mouse TBP) and loaded into Sapphire Chips (Stilla Technologies, Beverly, MA, USA). The Naica Geode was programed to perform the sample partitioning step followed by the PCR thermal cycling program: 95°C for 10 min, followed by 45 cycles of 95°C for 30 s and 60°C for 1 min. Image acquisition was performed using the Naica Prism3 reader using exposure times of 50 ms for the blue channel and 150 ms for the green channel. Total droplet enumeration and droplet quality control, enabled by the detection of the reference dye FITC in the blue channel, was performed by the Crystal Reader software. Extracted fluorescence values for each droplet were then further analyzed using the Crystal Miner software (Stilla Technologies, Beverly, MA, USA). GFP copy number was measured in the FAM channel (blue). Glucagon or TATA-binding protein (TBP) copy numbers were measured in the VIC channel (green).

### Circular AAV DNA quantification assay

Extracted DNA for each sample was diluted to 20 ng per μl. A total of 100 ng of DNA for each sample was digested with PS-DNase (Lucigen, Middleton,WI, USA) to isolate circular DNA by removing any linear DNA within the sample. The reaction mix consisted of 100 ng of DNA, 10 units of PS-DNase, 33 mM Tris-acetate (pH 7.5), 66 mM potassium acetate, 10 mM of magnesium acetate, 0.5 mM DTT, and 1 mM ATP. DNA samples were incubated with PS-DNase at 37°C for 16 h, and then heat inactivated for 30 min at 70°C. A reaction with the same amount of DNA but no PS-DNase was performed simultaneously as a control. Digested and control samples were diluted 10-fold and 5 μl of the diluted sample was used for ddPCR. The number of circular DNA copies per diploid genome was quantified using the BioRad QX200 ddPCR system. Primers and probe for GFP were used to detect number of circular DNA copies and mouse glucagon was used to normalize by diploid genome. To detect the number of AAV genomes within a circular format, an additional restriction enzyme digestion using ECORI was added to the ddPCR workflow. ECORI digestion breaks up concatamers containing multiple copies of the AAV genome into individual vector genomes by only cleaving once in each genome, allowing for accurate quantification of AAV genomes regardless of concatemer size. Five μl of the PS-DNase digested samples were added to the BioRad ddPCR reaction mixture containing an additional 5 units of ECORI enzyme. Reactions were incubated at 37°C for 30 min before continuing to droplet generation and PCR. Copies of AAV genomes were again quantified using GFP primers and probes, then normalized by copies of mouse glucagon.

### Statistics

All statistical analyses were performed using GraphPad Prism software. Data are presented at mean ± standard error of the mean. Each group consisted of 4 mice except for the 6-month CAG group as one mouse had to be prematurely euthanized due to severe dermatitis.

## Results

### Comparison of long-term transgene expression from different promoters in the striatum

**Figure 2:**
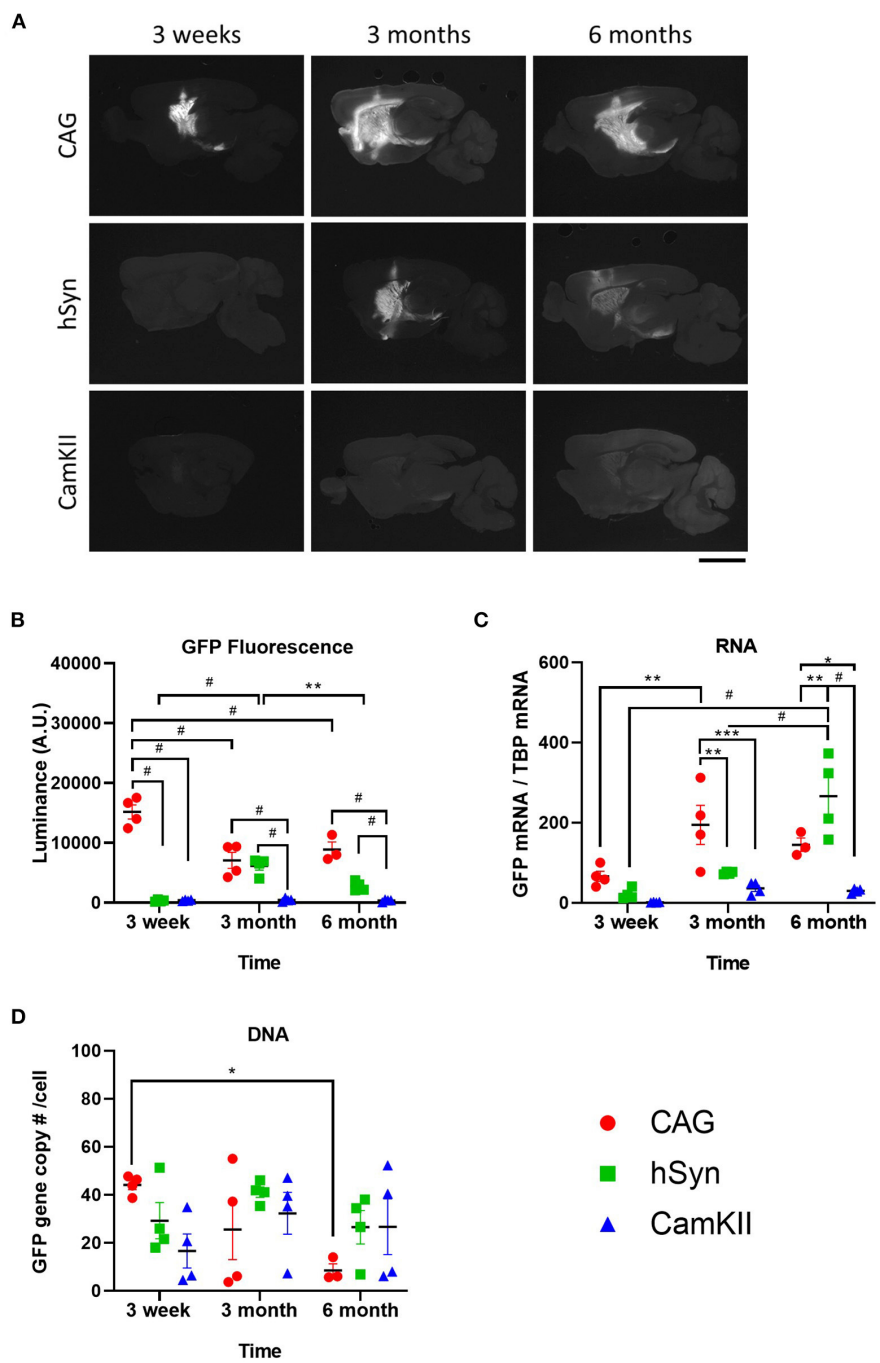
Long-term kinetics of rAAV9 genomes, transcripts and protein expression in the striatum in mice stereotaxically injected with rAAV9-CAG-GFP, rAAV9-hSyn-GFP, or rAAV9-CamKII-GFP. **(A)** Images and **(B)** quantification of luminance of native GFP fluorescence (500 ms exposure) at 3 weeks, 3 and 6 months after rAAV9-GFP administration. Scale bar represents 3 mm. **(C)** Amount of GFP mRNA relative to TBP mRNA and **(D)** GFP DNA copies per cell as determined by copy of GFP to two glucagon copies per cell. Data was analyzed using a two-way ANOVA followed by Tukey's multiple comparisons test. *p < 0.05, **p < 0.01, ***p < 0.001, and ^#^*p* < 0.0001.

Restricting transgene expression to neurons is frequently desirable, but the long-term activity of neuron-specific promoters has been suggested to be repressed or inactivated (14, 26). Therefore, the durability of expression of GFP driven by the neuron-specific promoters, hSyn and CamKII, was examined after intraparenchymal injection into the striatum of mice in comparison with the ubiquitous promoter, CAG. Groups of mice (*n*=4) were injected with rAAV9 encoding GFP under the CAG, hSyn, or CamKII promoters and transgene expression was examined over the course of 6 months (Figure 2). Fluorescent luminance (from the native fluorescence) was assessed as a proxy for GFP protein expression and was found to significantly change over the course of time depending on the promoter [Figures 2A,B; two-way ANOVA, *F*_(4,26)_ (time × promoter) = 22.68, *p* < 0.0001]. The greatest fluorescent luminance was produced by the CAG promoter at 3 weeks, followed by a decrease at 3 months, which then remained the same from 3 to 6 months. GFP luminance driven by the hSyn promoter was significantly lower than CAG at 3 weeks but increased to similar levels as CAG at 3 months. However, at 6 months, the luminance in the hSyn group decreased compared to hSyn expression at 3 months. Luminance in the striatum of mice injected with rAAV9-CamKII-GFP was significantly lower than CAG or hSyn over the entire time course.

GFP mRNA expression was also found to significantly change over the course of time depending on the promoter [Figure 2C; two-way ANOVA, *F*_(4,26)_ (time × promoter) = 25.77, *p* = 0.0002]. In contrast with the protein levels, GFP mRNA was not significantly different between groups at 3 weeks, but at 3 months the GFP mRNA in the CAG group was significantly increased compared with hSyn and CamKII. At 6 months, GFP mRNA in the hSyn group reached its peak, showing significantly higher expression compared to both the CAG and CamKII groups.

From 3 weeks to 6 months, there were no significant differences in the total amount of vector DNA across promoters [Figure 2D; two-way ANOVA, *F*_(4,26)_ (time × promoter) = 24.25, *p* = 0.0723]. There was, however, a significant decrease from 3 weeks to 6 months in the CAG group.

**Figure 3:**
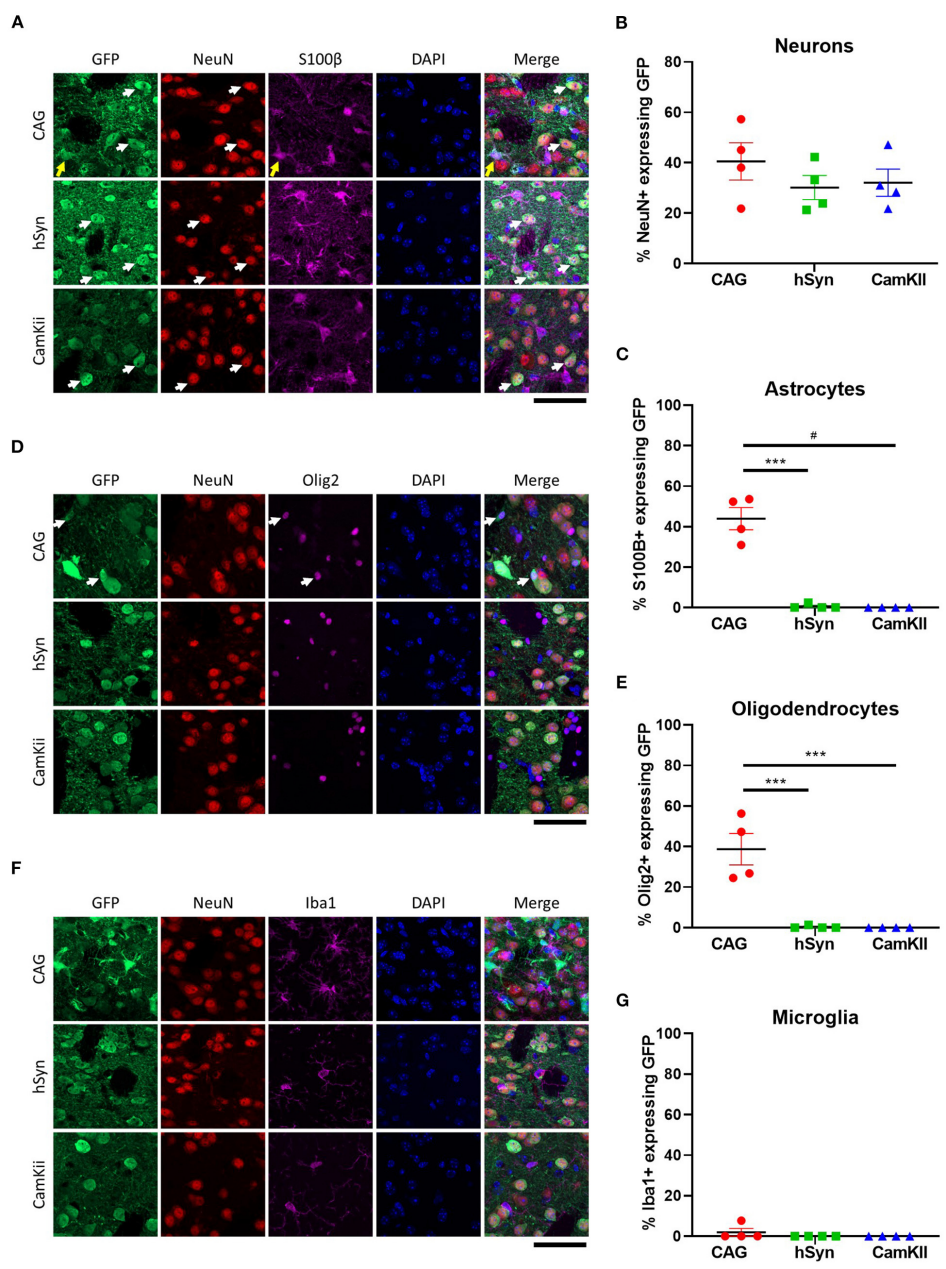
Cell-type specificity of GFP expression in the striatum of mice. **(A)** Colocalization of GFP expression with markers of neurons [NeuN] and astrocytes [S100β]. Arrows point out cells with co-localization of GFP with NeuN (white) and S100B (yellow). Percentage of **(B)** neurons and **(C)** astrocytes that express GFP in the transduced area. **(D)** Colocalization of GFP expression with markers of oligodendrocytes (Olig2) and **(E)** percentage of oligodendrocytes that express GFP in the transduced area. White arrows point out cells with co-localization of GFP with Olig2. **(F)** Colocalization of GFP expression with markers of microglia (Iba1) and **(G)** percentage of microglia that express GFP in the transduced area. Scale bar represents 50 μm. Data was analyzed using a one-way ANOVA followed by Tukey's multiple comparisons test. ***p < 0.001 and ^#^*p* < 0.0001.

To confirm that the neuronal promoters (hSyn and CamKII) primarily restricted GFP expression to neurons, immunohistochemistry was performed with cellular-type specific antibodies on tissue from the 3-week time point. Approximately 30–40% of neurons (NeuN-positive) expressed GFP in all groups [Figures 3A,B; one-way ANOVA, *F*_(2,9)_ = 0.8651, *p* = 0.4533]. GFP expression driven by the hSyn or CamKII promoter were primarily restricted to neurons in contrast with the CAG promoter, which drove GFP expression in approximately 40% of astrocytes (S100β-positive) [Figures 3A,C; one-way ANOVA, *F*_(2,9)_ = 62.81, *p* < 0.0001] and oligodendrocytes [Olig2-positive; Figures 3D,E; one-way ANOVA, *F*_(2,9)_ =24.56, *p* = 0.0002]. GFP was not expressed in microglia [Iba1-positive; Figures 3F,G; one-way ANOVA, *F*_(2,9)_ =1.000, *p* = 0.4053] with the exception of scant expression in microglia in the CAG group.

### Temporal dynamics of vector genome processing and transgene expression driven by the CAG promoter in the mouse striatum

**Figure 4:**
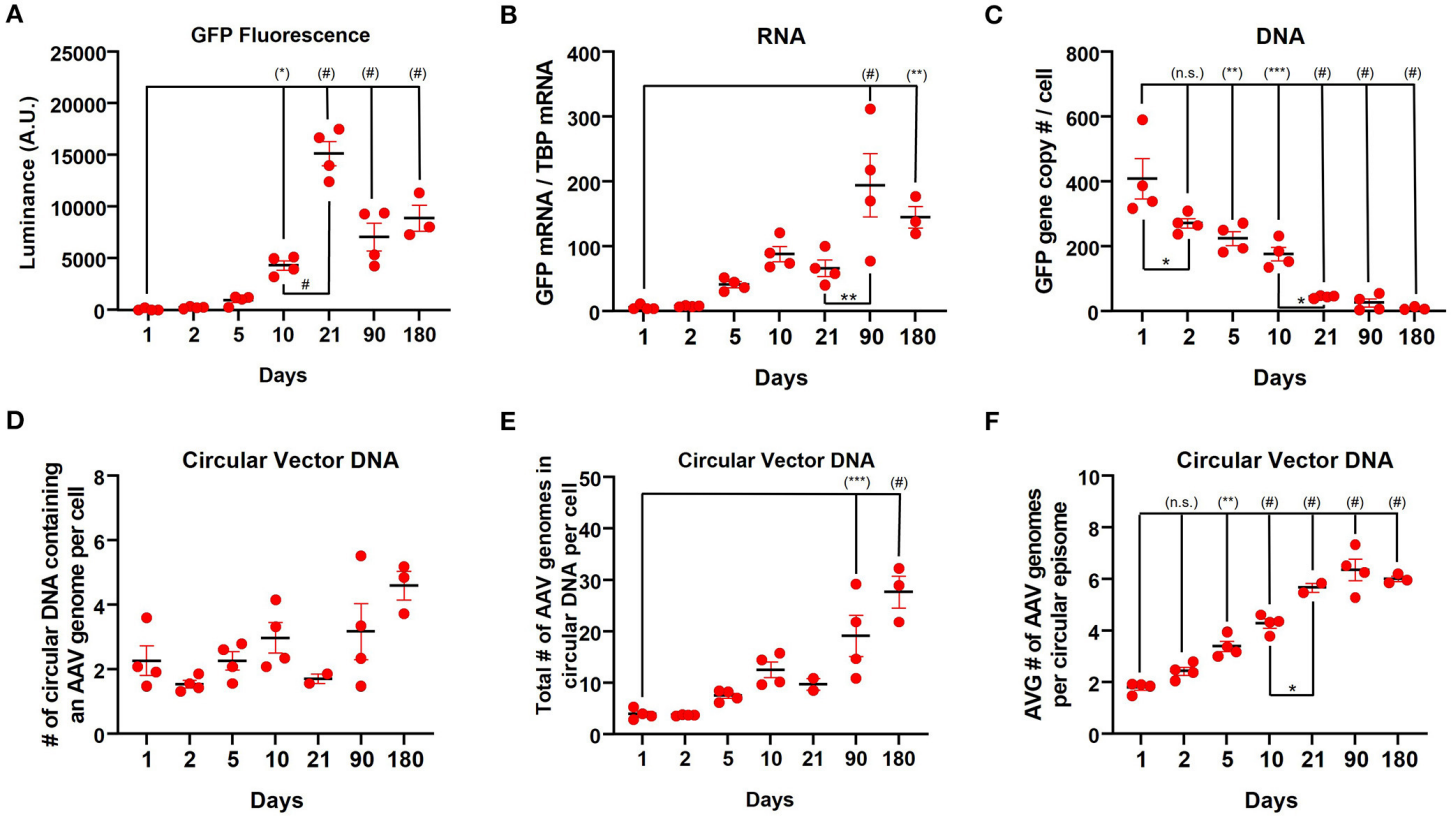
Long-term kinetics of rAAV9-CAG-GFP in the striatum in mice. **(A)** Quantification of luminance from native GFP fluorescence (500 ms exposure) during the first 6 months after rAAV9-CAG-GFP administration. **(B)** ddPCR analysis measuring the amount of GFP mRNA relative to TBP mRNA and **(C)** GFP DNA copies per cell as determined by copy numbers of GFP to two glucagon copies per cell during the first 6 months after administration of rAAV9-CAG-GFP. Data from 3 weeks, 3 and 6 months was previously presented in Figure 2. **(D)** The number of circular DNA units that contain an AAV genome (not considering concatemers) per cell, **(E)** the total number of AAV genomes in circular DNA per cell (diploid genome) and **(F)** the average number of AAV genomes per circular episome [values from **(E)** divided by values from **(D)**] in the striatum of mice during the first 6 months after administration of rAAV9-CAG-GFP. Data was analyzed using a one-way ANOVA followed by Tukey's multiple comparisons test. *p < 0.05, **p < 0.01, ***p < 0.001, and ^#^*p* < 0.0001. Significance symbols in parentheses are compare with day 1.

Based on the observation of very high GFP protein expression with relatively low RNA expression at 3 weeks post-injection observed with the CAG promoter, we next sought to characterize transgene expression during the first 10 days after vector administration. Following injection of rAAV9-CAG-GFP into the striatum of mice (*n*=4), the transgene (GFP) expression was assessed at 1, 2, 5, and 10 days after injection by measuring fluorescent luminance (proxy for protein levels), GFP mRNA and vector DNA. GFP expression could be detected by immunohistochemistry for GFP as early as 2 days post-administration (Supplementary Figure S2) and the fluorescent luminance increased until 3 weeks [Figure 4A; one-way ANOVA, *F*_(6,20)_ = 48.53, *p* < 0.0001]. The amount of GFP mRNA increased over the first 3 months followed by a decrease at 6 months [Figure 4B; one-way ANOVA, *F*_(6,20)_ = 11.15, *p* < 0.0001]. The amount of vector DNA was the inverse of GFP mRNA and fluorescent luminance with vector genomes rapidly declining from day 1 (~400 gc/cell) to 3 weeks (~40 gc/cell) after rAAV9 administration [Figure 4C; one-way ANOVA, *F*_(6,20)_ = 27.09, *p* < 0.0001].

Previous reports have demonstrated that there is a significant loss of vector DNA over the course of the first several weeks after rAAV administration in the liver (27) as the genomes form stable episomes; however, to our knowledge, this had not been examined in the brain. Therefore, the number of circular DNA units that contains an AAV genome per cell was assessed (Figure 4D and Supplementary Figure S3). Over the course of 6 months, the number of circular DNA units of containing AAV genomes per cell slightly increased [Figure 4D; one-way ANOVA, *F*_(6,18)_ =3.718, *p* = 0.0140]. Consistently, the total number of vector genomes in a unit of circular DNA per cell increased over this period [Figure 4E; one-way ANOVA, *F*_(6,18)_ =16.15, *p* < 0.0001]. Furthermore, the average number of vector genomes per unit of circular DNA increased over this time period [Figure 4F; one-way ANOVA, *F*_(6,18)_ =59.58, *p* < 0.0001] indicating that the average size of episomes of AAV genomes is increasing over time. Over the course of 3 weeks to 6 months, CAG, hSyn, and CamKII showed similar trends with the number of circular DNA units containing AAV genomes per cell (Supplementary Figure S4A) and the total number of vector genomes in circular DNA per cell (Supplementary Figure S4B) both increasing. However, the average number of vector genomes per unit of circular DNA remained constant over this time (Supplementary Figure S4C).

## Discussion

**Figure 5:**
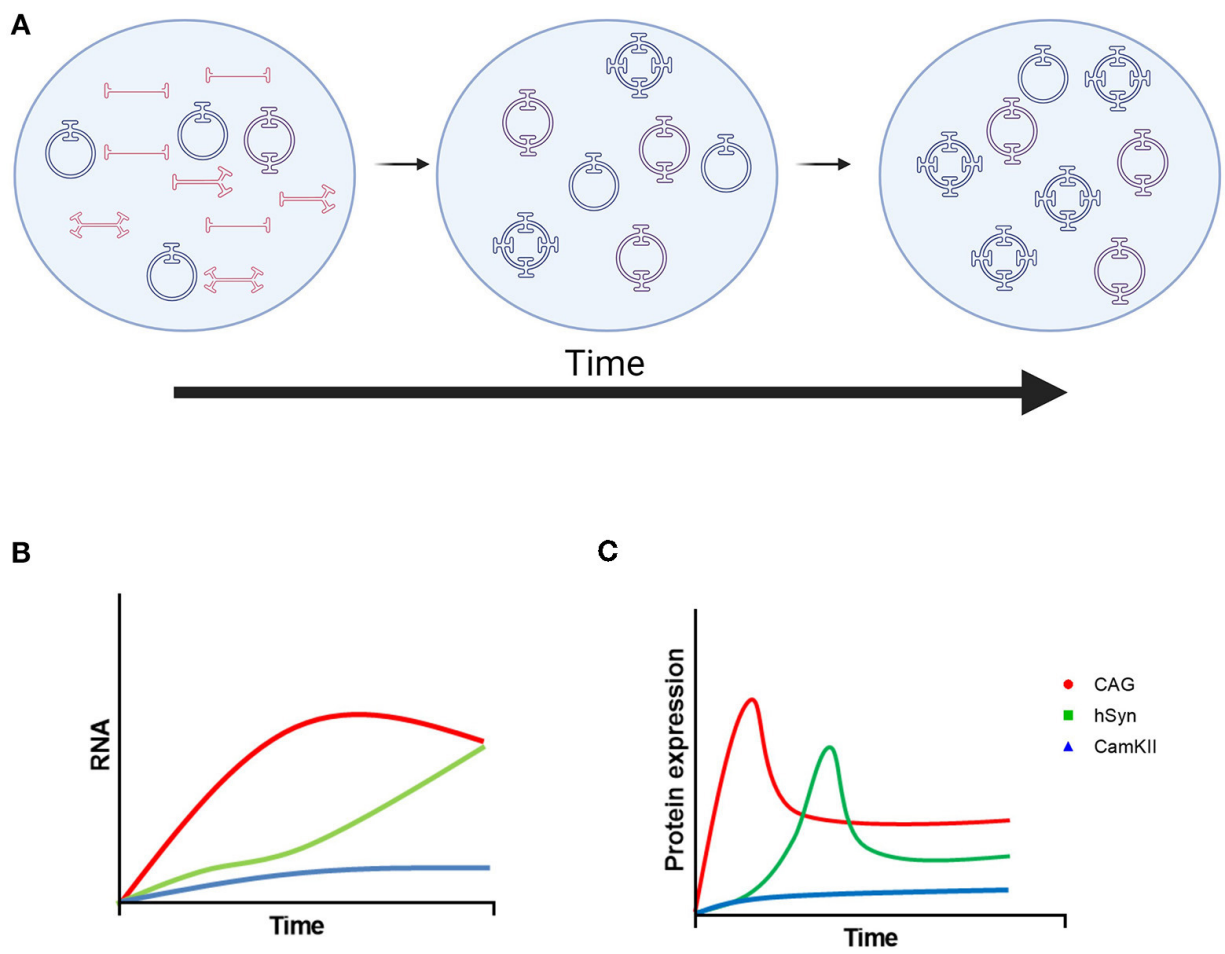
Model of rAAV9 genome processing and transgene expression. **(A)** Linear single-stranded rAAV DNA is transported to the nucleus and then converted to linear double-stranded genomes by second-strand synthesis or strand annealing. Linear double-stranded genomes are converted to stable, circular episomes as monomers or concatemers. Over time, the number of AAV genomes per circular episome increases until it becomes stable. The format of AAV DNA contributing to the increase in concatemer size is currently uncharacterized. **(B)** Proposed mRNA and **(C)** protein kinetics of transgenes driven by the CAG, hSyn, and CamKII promoters. Created using images from BioRender.com.

Here, we compared the ubiquitous CAG promoter with two neuron-specific promoters, hSyn and CamKII, in the striatum of adult mice after intraparenchymal injection over the course of 6 months. As expected, CAG provided a more robust and rapid expression of GFP in the mouse striatum with expression in neurons, astrocytes and oligodendrocytes. The hSyn promoter had significantly less expression compared with CAG at 3 weeks after vector administration, but it reached similar GFP expression to CAG at 3 months. The CamKII promoter provided neuron-specific GFP expression that was weak over the entire 6 months. These studies also provided insights into the kinetics and durability of transgene expression driven by the CAG, hSyn and CamKII promoters. In addition, these experiments demonstrated the formation of stable episomes as circular DNA in the CNS after intraparenchymal administration. A summary diagram of these kinetics is shown in Figure 5.

### Kinetics and durability of expression

The results for these studies with AAV9-CAG-GFP demonstrated the rapid expression of GFP, which was detectable by immunohistochemistry at day 2, and increased until 3 weeks in the mouse striatum. After 3 weeks, GFP expression decreased to a seemingly stable level at 3 and 6 months after administration. The GFP mRNA driven by CAG increased until it reached its peak at 3 months suggesting the promoter is still strong even though the protein expression declined relative to 3 weeks. In contrast with the expression driven by CAG, the CamKII promoter was much weaker over this timecourse, which was different from a recent report (20). One potential reason for the constant, low-level expression by CamKII relative to previous reports is that our vectors used in this study do not contain a WPRE element (as it is not used in clinical AAV therapeutics). GFP expression under control of hSyn reached its peak at 3 months and then declined at 6 months after administration. However, without additional late time points it is difficult to determine if the GFP expression reached a stable level. Despite the decrease in protein expression, the amount of GFP mRNA continued to increase at 6 months suggesting that the promoter is not being repressed or inactivated, but rather increases in strength over the entire 6 months.

A previous study found that GFP expression driven by the hSyn promoter decreased 22 weeks after intravenous rAAV9-hSyn-GFP relative to 4 weeks after administration in neonatal rats (14). Despite a systemic administration, only the cerebellum was analyzed for expression and only by fluorescence providing little insight into the numerous possibilities that could be affecting the loss of fluorescence. Here, we found that GFP expression driven by the hSyn promoter drove strong expression 3 months after direct intracranial rAAV9 injection into the striatum, but GFP expression was still strongly present at 6 months. In contrast to the decrease in protein expression as measured by luminance, the amount of GFP mRNA significantly increased at 6 months compared with 3 months and 3 weeks after vector administration. Importantly, the amount of vector DNA remained the same from 3 weeks to 6 months. These data suggest that the promoter is still active, and still increasing in strength, but there is a decoupling of mRNA levels with GFP protein expression. The mechanisms resulting in decoupling of mRNA levels with GFP protein expression could be decreasing translation after transcription, post-translational modifications to GFP protein, or an increase in GFP turnover rate as the cells respond to overexpression of GFP. Therefore, this decrease in GFP protein expression may be GFP specific. Future studies with expression of endogenous proteins or other transgene products, e.g., antibodies or microRNAs, would be informative to assess whether these readouts apply to establishing stable expression in different contexts.

The translational aspects of examining GFP protein expression may be limited, but it does provide critical insights into the biology and kinetics of transgenes delivered by rAAV in the brain after intraparenchymal delivery as successful and durable transgene expression involves multiple, complex processes. Once the single-stranded, linear vector DNA is transported to the nucleus, second strand synthesis occurs resulting in double-stranded, linear genomes (1). Double-strand break repair yields stable, circular episomes that can be monomeric or concatemeric and are associated with long-term transgene expression (27). Measuring the GFP gene copy number per cell by ddPCR during the first 3 weeks found that the amount of vector DNA decreased as previously reported in the liver (27). However, PS-DNase and EcoRI treatment of DNA from the striatum demonstrated that the total number of AAV genomes in circular DNA form increased over the first 3 weeks even though the number of circular DNA units that contain an AAV genome (excluding the number of AAV genomes copies per circular DNA unit) largely remained similar. In addition, the average number of AAV genome copies per circular piece of DNA increased as well over the first 3 weeks. Taken together, these findings are consistent with more vector genomes being degraded during the first 3 weeks after injection and only a small portion of genomes resulting in stable, circular episomes (Figure 5). The average number of AAV genomes per circular DNA unit gradually increased over the entire 6 months suggesting that more vector genomes are being incorporated into stable episomes in monomeric or concatemeric forms. The type of AAV DNA that contributes to these increases are currently unclear and require further investigation. However, these studies provide novel insight into AAV genome-processing for delivered rAAVs in the brain.

In addition, understanding how the kinetics of transgenes driven by different promoters contributes to the intended effects will be vital to gene therapy development in the CNS. For example, if the transgene modulates a neural circuit, it may be more beneficial to use the hSyn promoter that has slower initial kinetics of expression, particularly in adults where the brain is less plastic to larger changes (28). Here, the lower increment of transgene expression may allow for a larger capacity of plasticity of the neural circuits being modulated. The comparatively rapid and robust transgene expression driven by CAG may then limit the achievable effect on neural circuits by trying to modulate the circuit too quickly. While this is speculative, understanding these dynamics would be critical for gene therapy development.

### Cell-type specificity of promoters

As demonstrated in Figure 3, transgene expression was primarily restricted to neurons in the striatum by using the neuron-specific promoters, hSyn and CamKII, to drive GFP expression in comparison to the CAG promoter driving GFP expression in a significant proportion of neurons, astrocytes and oligodendrocytes. While this study examines transgene expression in the striatum as a whole, it does not examine the strength of transgene expression in different neuronal cell types, i.e., direct vs. indirect medium spiny neurons, parvalbumin vs. cholinergic interneurons, etc., which is best resolved in a future study. Furthermore, it would be interesting to assess how the lack of promoter activity in non-neuronal cells contribute to the overall readouts of mRNA and luminance. There may be different accumulation of the number vector genomes per cell or different efficiencies of episome/concatemer formation in different cell types as well as different transduction efficiencies of various cell types. Future studies with cell sorting or combinations of *in situ* hybridization with IHC to identify cell-type could offer insights into these differences.

### Applications to basic and translational science

The understanding of transgene expression and kinetics are not only vital in the long-term for gene therapies, but it is also vital for the design and analysis of basic neuroscience studies. Our results demonstrate that transgene expression continues to change in mice until at least 3 months depending on promoter. Therefore, differences in transgene expression could confound results if a response is recorded over a long period of time making the inclusion of experimental controls paramount. For example, channel rhodopsin expression under the CAG promoter could be dramatically different on day 10 compared to day twenty-one, likely resulting in stronger modification of neuronal output at the later time point (even with comparable laser power), and thus yield different behavioral output. This could be particularly problematic for studies of reinforcement learning for example, where optogenetic self-stimulation paradigms (e.g., of dopamine neurons) occurring over several concurrent days or weeks show animals increase lever pressing to self-stimulate their dopamine neurons over time. One interpretation is that the animal is learning, but our results alternatively would suggest that the increased opsin expression is making the stimulation intrinsically more reinforcing. Caution is warranted in the interpretation of results relying on AAV-based expression that has been demonstrated here to fluctuate over time.

## Data availability statement

The original contributions presented in the study are included in the article/Supplementary material, further inquiries can be directed to the corresponding author.

## Ethics statement

The animal study was reviewed and approved by IACUC at CL Laboratory LLC.

## Author contributions

BH performed the *in vivo* studies, ddPCR experiments, and wrote the manuscript. HC and MF sectioned tissues, performed IHC with analysis, and luminance analysis. RQ designed and performed the circular DNA assays. AG designed and performed supporting *in vitro* experiments. AM, OD, YL, and JB contributed to experimental design and interpretation of data. JS conceived of the project, contributed to experimental design, interpretation of data, and writing of the manuscript. All authors contributed to the article and approved the submitted version.

## Funding

This study research was funded entirely by REGENXBIO Inc.

## Conflict of interest

This study received funding from REGENXBIO Inc. The funder had the following involvement in the study. All authors are employees of REGENXBIO Inc. and thus designed the studies, performed the experiments, analyzed the data, generated the figures, and wrote the manuscript.

## Publisher's note

All claims expressed in this article are solely those of the authors and do not necessarily represent those of their affiliated organizations, or those of the publisher, the editors and the reviewers. Any product that may be evaluated in this article, or claim that may be made by its manufacturer, is not guaranteed or endorsed by the publisher.

## Supplementary material

Supplementary Figure 1 Exposure series of the luminance of native GFP expression in the striatum of rAAV9-CAG-GFP injected mice to determine optimal exposures for quantification of luminance. **(A)** Quantification and **(B)** representative images of the native fluorescence from GFP expression imaged with 100, 500, and 1,000 ms exposures in the striatum of mice 5 days, 10 days, 21 days, 3 months, and 6 months after being injected with rAAV9-CAG-GFP. Luminance values represented in arbitrary units (A.U.). Scale bar represents 3 mm.

Supplementary Figure 2 GFP expression detected by immunohistochemistry for GFP at 1 and 2 days after rAAV9-CAG-GFP administration. GFP expression was visibly detectable on post-injection day 2.

Supplementary Figure 3 Diagram depicting the circular AAV genome quantification assay. Accurate measurement of AAV genomes within the context of an AAV genome population that is mostly circular requires ECORI digestion. For PSDNASE only digested samples, a concatemer with multiple AAV genomes will count for the same number of positive droplets as a monomer as ddPCR only measures the end point of the PCR reaction. However, the addition of a restriction enzyme digests concatemers into multiple linear AAV genomes, thereby increasing the positive droplets to the corresponding number of AAV genomes released from a concatemer. Created using images from BioRender.com.

Supplementary Figure 4 Long-term kinetics of rAAV9 concatemers in the striatum in mice. **(A)** The number of circular DNA units that contain an AAV genome (not considering concatemers) per cell, **(B)** the total number of AAV genomes in circular DNA per cell (diploid genome) and **(C)** the average number of AAV genomes per circular episome [values from **(B)** divided by values from **(A)**] in the striatum of mice during the first 6 months after administration of rAAV9-CAG-GFP, rAAV9-hSyn-GFP, and rAAV9-CamKII-GFP.

Supplementary Table 1 Promoter sequences used in these studies.
