# Supplementary figures and images for: Kinetics and durability of transgene expression after intrastriatal injection of AAV9 vectors
Source: Front Neurol. 2022 Nov 14;13:1051559. doi: 10.3389/fneur.2022.1051559 (PMC9702554; doi:10.3389/fneur.2022.1051559)

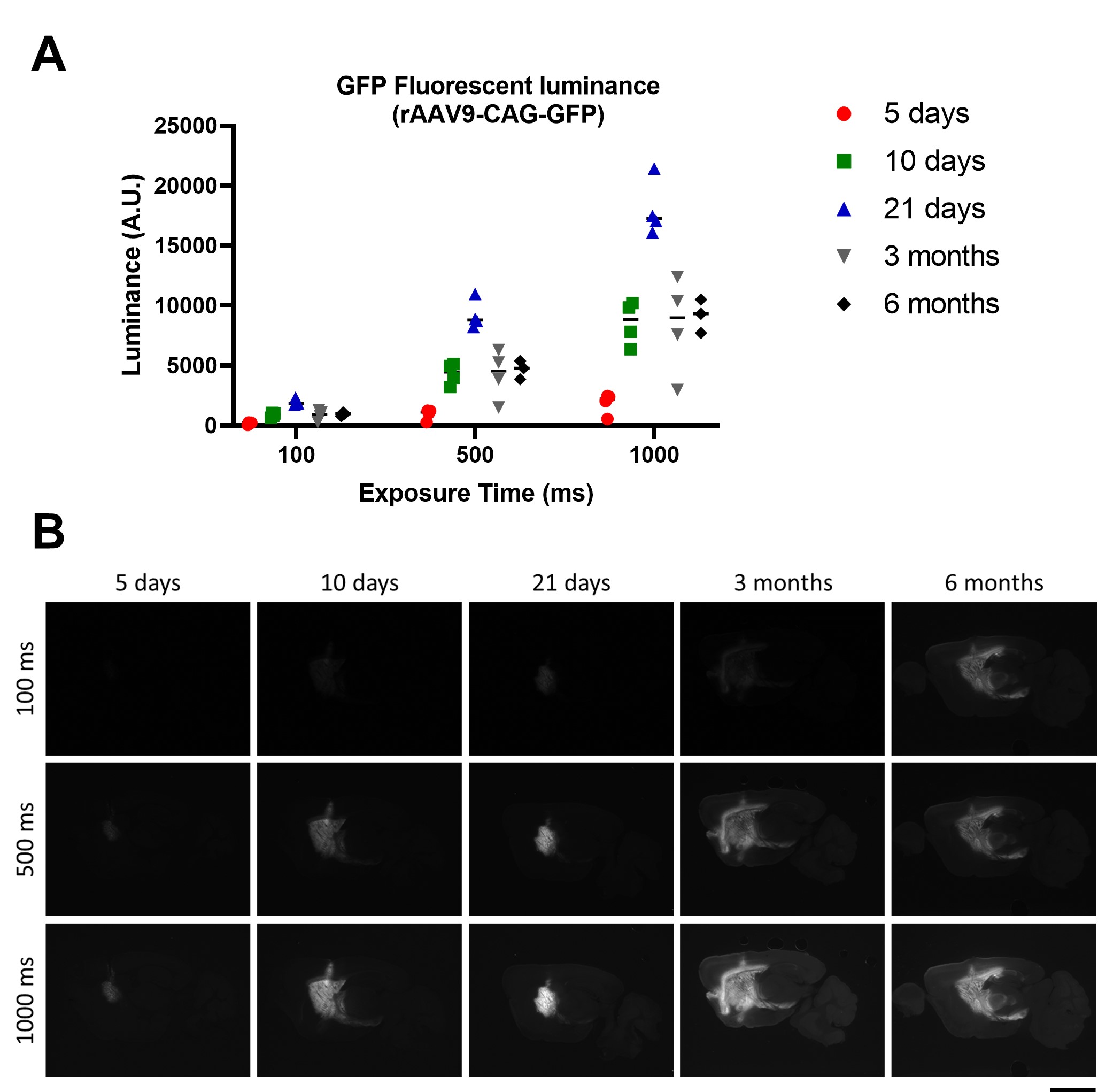

Supplement: Supplementary Figure 1 — Exposure series of the luminance of native GFP expression in the striatum of rAAV9-CAG-GFP injected mice to determine optimal exposures for quantification of luminance. (A) Quantification and (B) representative images of the native fluorescence from GFP expression imaged with 100, 500, and 1,000 ms exposures in the striatum of mice 5 days, 10 days, 21 days, 3 months, and 6 months after being injected with rAAV9-CAG-GFP. Luminance values represented in arbitrary units (A.U.). Scale bar represents 3 mm. [file Image_1.JPEG]

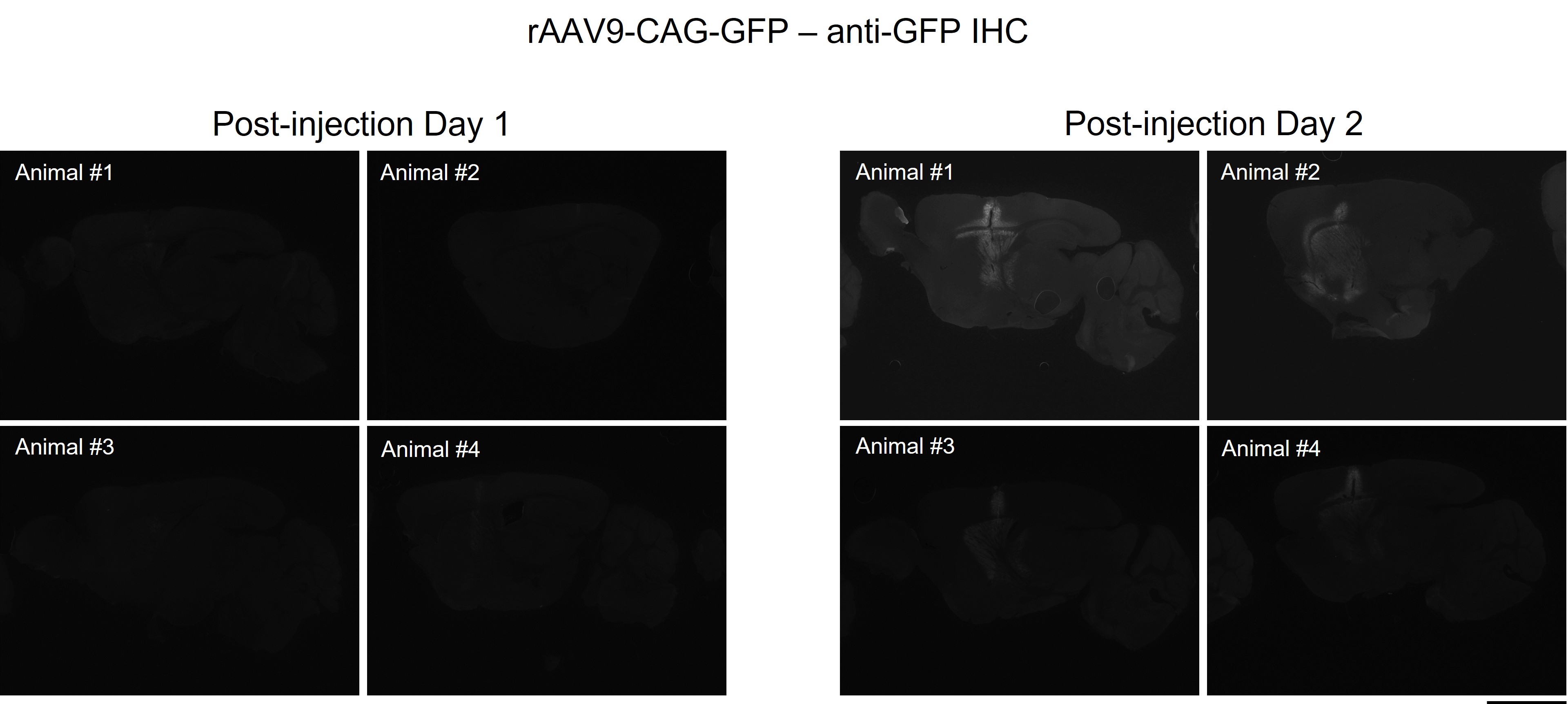

Supplement: Supplementary Figure 2 — GFP expression detected by immunohistochemistry for GFP at 1 and 2 days after rAAV9-CAG-GFP administration. GFP expression was visibly detectable on post-injection day 2. [file Image_2.JPEG]

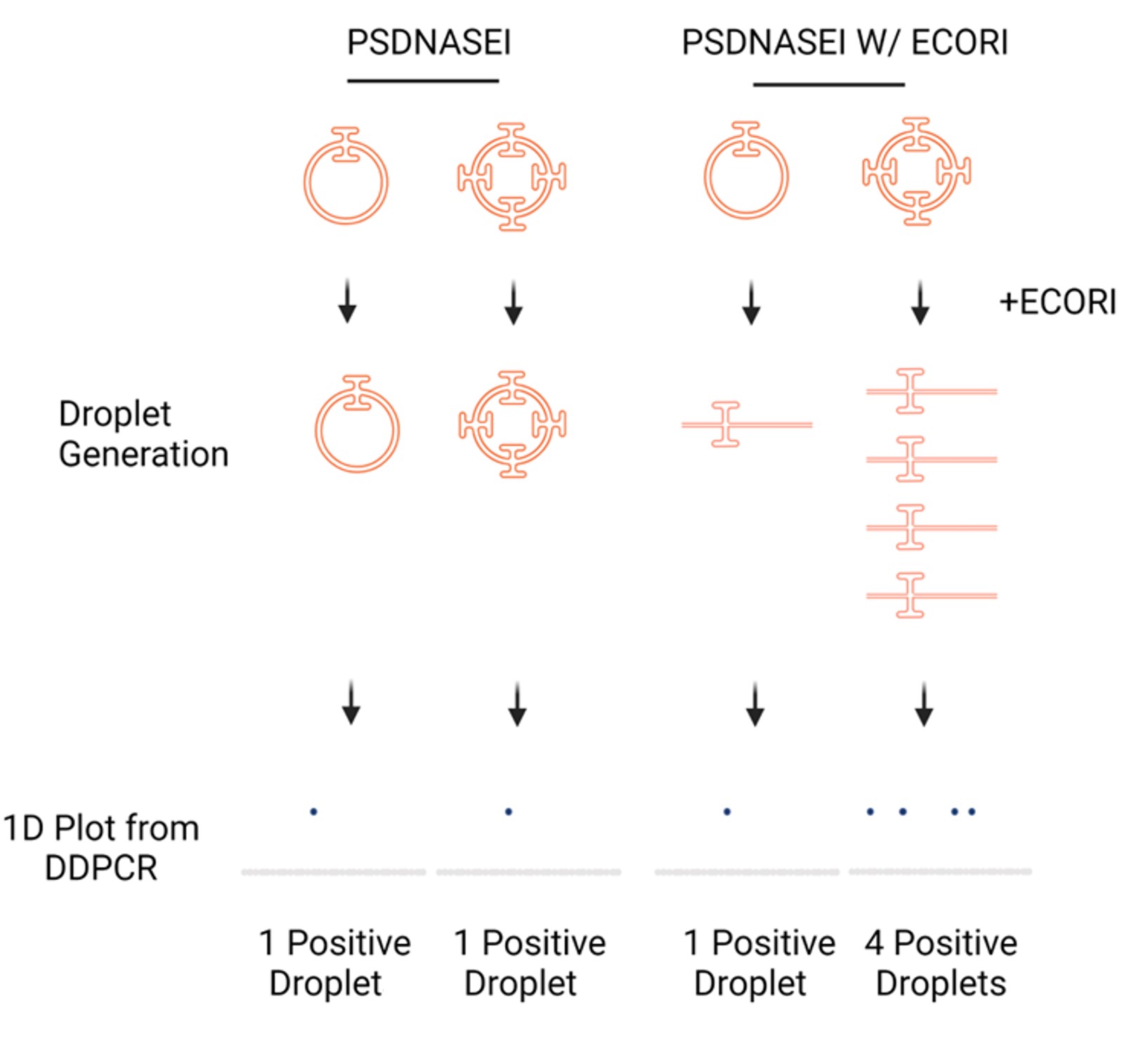

Supplement: Supplementary Figure 3 — Diagram depicting the circular AAV genome quantification assay. Accurate measurement of AAV genomes within the context of an AAV genome population that is mostly circular requires ECORI digestion. For PSDNASE only digested samples, a concatemer with multiple AAV genomes will count for the same number of positive droplets as a monomer as ddPCR only measures the end point of the PCR reaction. However, the addition of a restriction enzyme digests concatemers into multiple linear AAV genomes, thereby increasing the positive droplets to the corresponding number of AAV genomes released from a concatemer. Created using images from BioRender.com. [file Image_3.JPEG]

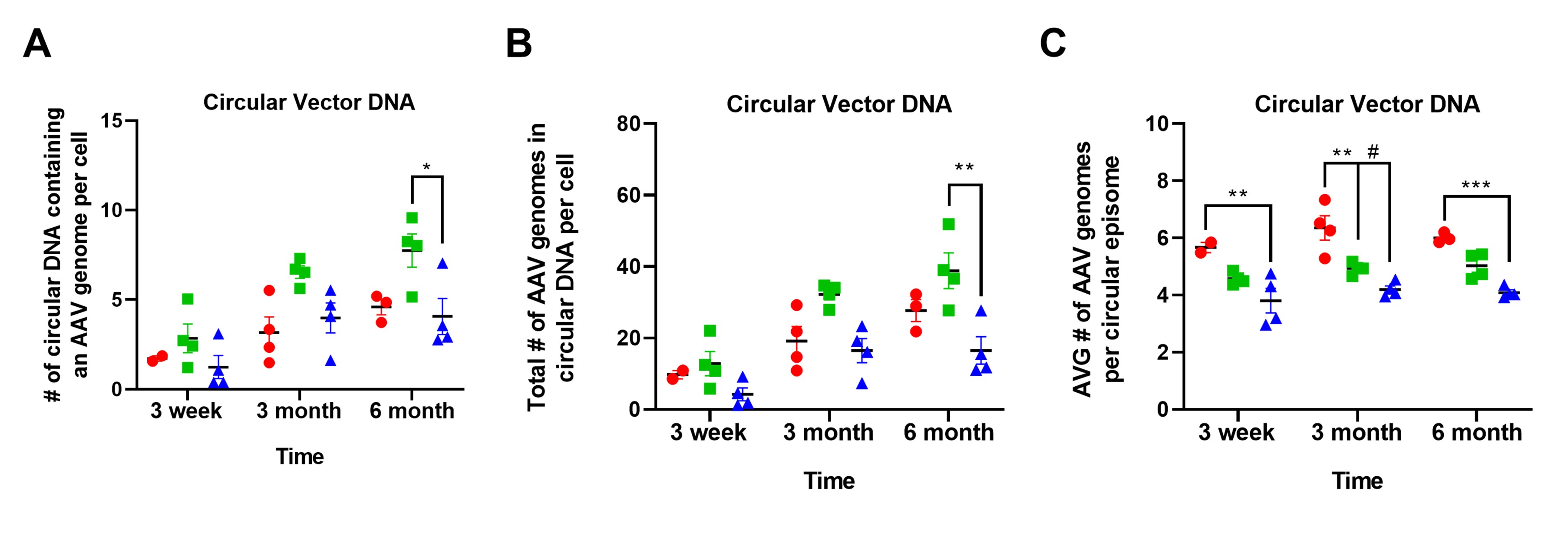

Supplement: Supplementary Figure 4 — Long-term kinetics of rAAV9 concatemers in the striatum in mice. (A) The number of circular DNA units that contain an AAV genome (not considering concatemers) per cell, (B) the total number of AAV genomes in circular DNA per cell (diploid genome) and (C) the average number of AAV genomes per circular episome [values from (B) divided by values from (A)] in the striatum of mice during the first 6 months after administration of rAAV9-CAG-GFP, rAAV9-hSyn-GFP, and rAAV9-CamKII-GFP. [file Image_4.JPEG]
