# Supplementary material for: Kinetics and durability of transgene expression after intrastriatal injection of AAV9 vectors
Source: Front Neurol. 2022 Nov 14;13:1051559. doi: 10.3389/fneur.2022.1051559 (PMC9702554; doi:10.3389/fneur.2022.1051559)
Supplement: Supplementary Table 1 — Promoter sequences used in these studies. [file Table_1.pdf]

| Promoter | Sequence |
|---|---|
| CAG | GACATTGATTATTGACTAGTTATTAATAGTAATCAATTACGGGGTCATTAGTTCATAGCCCATATATGGAGTTCCGCGTTACATAACTTACGGTAAATGGCCCGCCTGGCTGACCGCCCAACGACCCCCGCCCATTGACGTCAA<br>TAATGACGTATGTTCCCATAGTAACGCCAATAGGGACTTTCCATTGACGTCAATGGGTGGAGTATTTACGGTAAACTGCCCACTTGGCAGTACATCAAGTGTATCATATGCCAAGTACGCCCCCTATTGACGTCAATGACGGTA<br>AATGGCCCGCCTGGCATTATGCCCAGTACATGACCTTATGGGACTTTCCTACTTGGCAGTACATCTACGTATTAGTCATCGCTATTACCATGGTCGAGGTGAGCCCCACGTTCTGCTTCACTCTCCCCATCTCCCCCCCCTCCCCA<br>CCCCCAATTTTGTATTTATTTATTTTTTAATTATTTTGTGCAGCGATGGGGGCGGGGGGGGGGGGGGGGCGCGCGCCAGGCGGGGCGGGGCGGGGCGAGGGGCGGGGCGGGGCGAGGCGGAGAGGTGCGGCGGCAGCC<br>AATCAGAGCGGCGCGCTCCGAAAGTTTCCTTTTATGGCGAGGCGGCGGCGGCGGCGGCCCTATAAAAAGCGAAGCGCGCGGCGGGCGGGAGTCGCTGCGCGCTGCCTTCGCCCCGTGCCCCGCTCCGCCGCCGCCTCGCG<br>CCGCCCGCCCCGGCTCTGACTGACCGCGTTACTCCCACAGGTGAGCGGGCGGGACGGCCCTTCTCCTCCGGGCTGTAATTAGCGCTTGGTTTAATGACGGCTTGTTTCTTTTCTGTGGCTGCGTGAAAGCCTTGAGGGGCTCC<br>GGGAGGGCCCTTTGTGCGGGGGGAGCGGCTCGGGGGGTGCGTGCGTGTGTGTGTGCGTGGGGAGCGCCGCGTGCGGCTCCGCGCTGCCCGGCGGCTGTGAGCGCTGCGGGCGCGGCGCGGGGCTTTGTGCGCTCCGCA<br>GTGTGCGCGAGGGGAGCGCGGCCGGGGGCGGTGCCCCGCGGTGCGGGGGGGGCTGCGAGGGGAACAAAGGCTGCGTGCGGGGTGTGTGCGTGGGGGGGTGAGCAGGGGGTGTGGGCGCGTCGGTCGGGCTGCAACC<br>CCCCCTGCACCCCCCTCCCCGAGTTGCTGAGCACGGCCCGGCTTCGGGTGCGGGGCTCCGTACGGGGCGTGGCGCGGGGCTCGCCGTGCCGGGCGGGGGGTGGCGGCAGGTGGGGGTGCCGGGCGGGGCGGGGCCGCC<br>TCGGGCCGGGGAGGGCTCGGGGGAGGGGCGCGGCGGCCCCCGGAGCGCCGGCGGCTGTCGAGGCGCGGCGAGCCGCAGCCATTGCCTTTTATGGTAATCGTGCGAGAGGGCGCAGGGACTTCCTTTGTCCCAAATCTGT<br>GCGGAGCCGAAATCTGGGAGGCGCCGCCGCACCCCCTCTAGCGGGCGCGGGGCGAAGCGGTGCGGCGCCGGCAGGAAGGAAATGGGCGGGGAGGGCCTTCGTGCGTCGCCGCGCCGCCGTCCCCTTCTCCCTCTCCAGC<br>CTCGGGGCTGTCCGCGGGGGGACGGCTGCCTTCGGGGGGGACGGGGCAGGGCGGGGTTCGGCTTCTGGCGTGTGACCGGCGGCTCTAGAGCCTCTGCTAACCATGTTCATGCCTTCTTCTTTTTCCTACAGCTCCTGGGCAA<br>CGTGCTGGTTATTGTGCTGTCTCATCATTTTGGCAAAG |
| hSyn | AGTGCAAGTGGGTTTTAGGACCAGGATGAGGCGGGGTGGGGGTGCCTACCTGACGACCGACCCCGACCCACTGGACAAGCACCCAACCCCCATTCCCCAAATTGCGCATCCCCTATCAGAGAGGGGGAGGGGAAACAGGA<br>TGCGGCGAGGCGCGTGCGCACTGCCAGCTTCAGCACCGCGGACAGTGCCTTCGCCCCCGCCTGGCGGCGCGCGCCACCGCCGCCTCAGCACTGAAGGCGCGCTGACGTCACTCGCCGGTCCCCCGCAAACTCCCCTTCCCGG<br>CCACCTTGGTCGCGTCCGCGCCGCCGCCGGCCCAGCCGGACCGCACCACGCGAGGCGCGAGATAGGGGGGCACGGGCGCGACCATCTGCGCTGCGGCGCCGGCGACTCAGCGCTGCCTCAGTCTGCGGTGGGCAGCGGA<br>GGAGTCGTGTCGTGCCTGAGAGCGCA |
| CamKII | CATTATGGCCTTAGGTCACTTCATCTCCATGGGGTTCTTCTTCTGATTTTCTAGAAAATGAGATGGGGGTGCAGAGAGCTTCCTCAGTGACCTGCCCAGGGTCACATCAGAAATGTCAGAGCTAGAACTTGAACTCAGATTACT<br>AATCTTAAATTCCATGCCTTGGGGGCATGCAAGTACGATATACAGAAGGAGTGAACTCATTAGGGCAGATGACCAATGAGTTTAGGAAAGAAGAGTCCAGGGCAGGGTACATCTACACCACCCGCCCAGCCCTGGGTGAGT<br>CCAGCCACGTTCACCTCATTATAGTTGCCTCTCTCCAGTCCTACCTTGACGGGAAGCACAAGCAGAAACTGGGACAGGAGCCCCAGGAGACCAAATCTTCATGGTCCCTCTGGGAGGATGGGTGGGGAGAGCTGTGGCAGA<br>GGCCTCAGGAGGGGCCCTGCTGCTCAGTGGTGACAGATAGGGGTGAGAAAGCAGACAGAGTCATTCCGTCAGCATTCTGGGTCTGTTTGGTACTTCTTCTCACGCTAAGGTGGCGGTGTGATATGCACAATGGCTAAAAAG<br>CAGGGAGAGCTGGAAAGAAACAAGGACAGAGACAGAGGCCAAGTCAACCAGACCAATTCCCAGAGGAAGCAAAGAAACCATTACAGAGACTACAAGGGGGAAGGGAAGGAGAGATGAATTAGCTTCCCCTGTAAACCTT<br>AGAACCCAGCTGTTGCCAGGGCAACGGGGCAATACCTGTCTCTTCAGAGGAGATGAAGTTGCCAGGGTAACTACATCCTGTCTTTCTCAAGGACCATCCCAGAATGTGGCACCCACTAGCCGTTACCATAGCAACTGCCTCTT<br>TGCCCCACTTAATCCCATCCCGTCTGTTAAAAGGGCCCTATAGTTGGAGGTGGGGGAGGTAGGAAGAGCGATGATCACTTGTGGACTAAGTTTGTTCGCATCCCCTTCTCCAACCCCCTCAGTACATCACCCTGGGGGAACAG<br>GGTCCACTTGCTCCTGGGCCCACACAGTCCTGCAGTATTGTGTATATAAGGCCAGGGCAAAGAGGAGCAGGTTTTAAAGTGAAAGGCAGGCAGGTGTTGGGGAGGCAGTTACCGGGGCAACGGGAACAGGGCGTTTCGGA<br>GGTGGTTGCCATGGGGACCTGGATGCTGACGAAGGCTCGCGAGGCTGTGAGCAGCCACAGTGCCCTGCTCAGAAGCCCCAAGCTCGTCAGTCAAGCCGGTTCTCCGTTTGCACTCAGGAGCACGGGCAGGCGAGTGGCCCC<br>TAGTTCTGGGGGCAGC |
